# On the use of functional responses to quantify emergent multiple predator effects

**DOI:** 10.1038/s41598-018-30244-9

**Published:** 2018-08-06

**Authors:** Arnaud Sentis, David S. Boukal

**Affiliations:** 1 Unité Mixte de Recherche 5174 “Evolution et Diversité Biologique,” Centre National de la Recherche Scientifique - Université de Toulouse III - Institut de Recherche pour le Développement, 31062 Toulouse, France; 2 University of South Bohemia, Faculty of Science, Department of Ecosystem Biology, Branišovská 31a, 37005 České Budějovice, Czech Republic; 3 Czech Academy of Sciences, Biology Centre, Institute of Entomology, Branišovská 31, 37005 České Budějovice, Czech Republic; 4 Ecological Networks and Global Change Group, Experimental and Theoretical Ecology Station, UMR5321, CNRS - University Paul Sabatier, Moulis, France

## Abstract

Non-independent interactions among predators can have important consequences for the structure and dynamics of ecological communities by enhancing or reducing prey mortality rate through, e.g., predator facilitation or interference. The multiplicative risk model, traditionally used to detect these emergent multiple predator effects (MPEs), is biased because it assumes linear functional response (FR) and no prey depletion. To rectify these biases, two approaches based on FR modelling have recently been proposed: the direct FR approach and the population-dynamic approach. Here we compare the strengths, limitations and predictions of the three approaches using simulated data sets. We found that the predictions of the direct FR and the multiplicative risk models are very similar and underestimate predation rates when prey density is high or prey depletion is substantial. As a consequence, these two approaches often fail in detecting risk reduction. Finally, parameters estimated with the direct FR approach lack mechanistic interpretation, which limits the understanding of the mechanisms driving multiple predator interactions and potential extension of this approach to more complex food webs. We thus strongly recommend using the population-dynamic approach because it is robust, precise, and provides a scalable mechanistic framework to detect and quantify MPEs.

## Introduction

Determining the factors and mechanisms influencing the distribution and strengths of species interactions is crucial to understand and predict the dynamics and stability of ecological communities^1–5^. However, quantifying species interaction strengths (i.e., the effect of one species on the abundance of a second one, such as the effect of a predator on its prey) is challenging because species are embedded in complex communities and interaction strength between two species often depends on direct and indirect interactions with other species in the community. Observed community dynamics thus often differ from simple predictions based on pairwise interactions^6–8^. These differences emerge due to multiple-species interactions, often referred to (across various contexts) as higher-order interactions, trait-mediated indirect interactions, non-trophic interactions, and non-consumptive predator effects^7–10^.

Most previous studies of emergent multiple-species interactions have focused on multiple predator species with a shared prey^6,7,9,11^. They revealed that interactions among different predators (e.g., competition or intraguild predation), as well as phenotypic and behavioral responses of prey to predation risk, often lead to emergent multiple predator effects (MPEs), where prey consumption rates by multiple predators are inconsistent with the assumption of independent effects of each predator on prey survival^9,11^. MPEs are widespread in nature and can have profound effects on prey survival and transfer of energy and nutrients across trophic levels in food webs^12–14^. For instance, previous studies in agroecosystems showed that greater predator diversity can increase herbivore suppression and increase plant growth when predators facilitate each other^15^. The combined predation rate of multiple predators can sometime double the sum of their individual predation rates, showing that synergistic interaction among predators can be substantial and can improve biological control of agricultural pests^16^. Better understanding of these higher-order interactions is thus important for a full understanding of food web dynamics and ecosystem functioning^6,10,17^.

The most common approach used to quantify MPEs is to (1) experimentally estimate prey survival in the presence of each predator species in monoculture, (2) use this information to predict prey survival in the presence of multiple predator species using a mathematical model (hereafter; null model) assuming no emergent effects (i.e., predator species effects combine independently), and (3) compare null model predictions to empirical observations in which prey are exposed to a combination of the predator species. When predictions and observations do not differ, multiple predator species combine independently indicating the absence of emergent multiple predator effects. However, if predictions overestimate or underestimate observations, multiple predator species combine antagonistically (leading to prey risk reduction, where fewer prey are killed than predicted) or synergistically (leading to prey risk enhancement, where combined predators kill more prey than predicted by their individual effects). As the sign and magnitude of the differences among model predictions and empirical observations determine the nature and strength of MPEs, the accuracy and robustness of the null model predictions are crucial to detect and quantify MPEs.

Various null models have been proposed to predict the expected independent effects of multiple predators on prey survival^9,10,18–20^. A consensus emerged in the literature^19,21,22^ that the multiplicative risk model^18^ is the most accurate, and it has been widely used in multiple predator studies. The multiplicative risk model states that, if two predators have independent effects, the expected number of prey eaten by both predators can be predicted using the following equation:

1 $${N}_{{\rm{AB}}}={N}_{0}({P}_{{\rm{A}}}+{P}_{{\rm{B}}}-{P}_{{\rm{A}}}{P}_{{\rm{B}}})$$

where *N*_AB_ is the predicted number of prey eaten by predators A and B when foraging together, *N*_0_ is the initial number of prey, and *P*_*i*_ (*i* = A, B) is the probability of being eaten by predator *i*. However, recent studies argued against the use of the multiplicative risk model as it assumes constant prey mortality rate over the course of the experiment, which is only valid when predators have linear functional responses, when prey are continuously replenished (i.e. no prey depletion), or experimental durations are sufficiently short that depletion is inconsequential^23–25^. These assumptions are routinely violated in empirical studies of MPEs: functional responses of predators are nearly always a saturating function of prey density^26^ and prey depletion is often non-negligible. For example, McCoy *et al*.^13^ examined 100 multiple predator studies reviewed by Vance-Chalcraft *et al*.^11^ and found that, on average, prey were depleted by 70% over the course of these experiments. A simulation study by McCoy *et al*.^13^ demonstrated that these violations can severely affect the interpretation of the results of MPE studies. In particular, the above limitations of the multiplicative risk model can bias results at high prey densities or when prey depletion is substantial^13,24^.

To overcome the biases of the multiplicative risk model, recent MPE studies have stressed the importance of relationships based on mechanistic models and experimental designs that incorporate a gradient of the key ecological variable, such as prey density, rather than simple factorial designs that do not account for time dependence and nonlinear interaction strengths^12–14^. In particular, the shape of the predator functional response (i.e., the relationship between the number of available prey and the number of prey eaten by a predator) and predator-prey dynamics have been identified as crucial to (1) adequately assess the presence and strengths of MPEs, (2) identify the mechanisms driving changes in species interactions, and (3) extrapolate the results to larger temporal and spatial scales^12,13^.

Two approaches following these ideas have recently been proposed to investigate emergent MPEs. Both are based on functional response modelling and aim to circumvent the limitations of the multiplicative risk model by accounting for non-linear predation rates and prey depletion. In the approach proposed by Wasserman *et al*.^20^ (hereafter; direct FR approach), the null model (to which predictions are compared with experimental observations involving multiple predators to estimate MPEs) combines a functional response model with the multiplicative risk model (eqn 1). In the approach proposed by McCoy *et al*.^13^ (hereafter; population-dynamic approach), the null model combines a functional response model with a population dynamic model. The population-dynamic approach accounts for prey depletion and non-linear feeding rates (see Material and Methods for more details) and thus produces unbiased predictions about independent effects of multiple predators on prey survival^13^. It can thus be considered as the reference null model to which other approaches can be compared.

The direct FR approach is also appealing, but it remains unclear if and when its predictions differ from the multiplicative risk model and whether the underlying assumptions of the direct FR approach are robust and do not suffer from the same biases as the multiplicative risk model. We argue that comparing the different models/approaches used to predict multiple predator independent effects on prey survival is an important issue for (1) correctly assigning the nature (risk reduction versus enhancement) and magnitude of emergent MPEs and (2) identifying modelling approaches that are best suited to quantify MPEs. The objective of this study was thus to compare the strengths and limitations of the multiplicative risk model, the direct FR model and the population dynamic model, and illustrate their use and compare their predictions using simulated data sets. We believe that this comparison will help advance the study of MPEs and improve the realism of predator-prey and food-web models.

## Results

**Figure 1:**
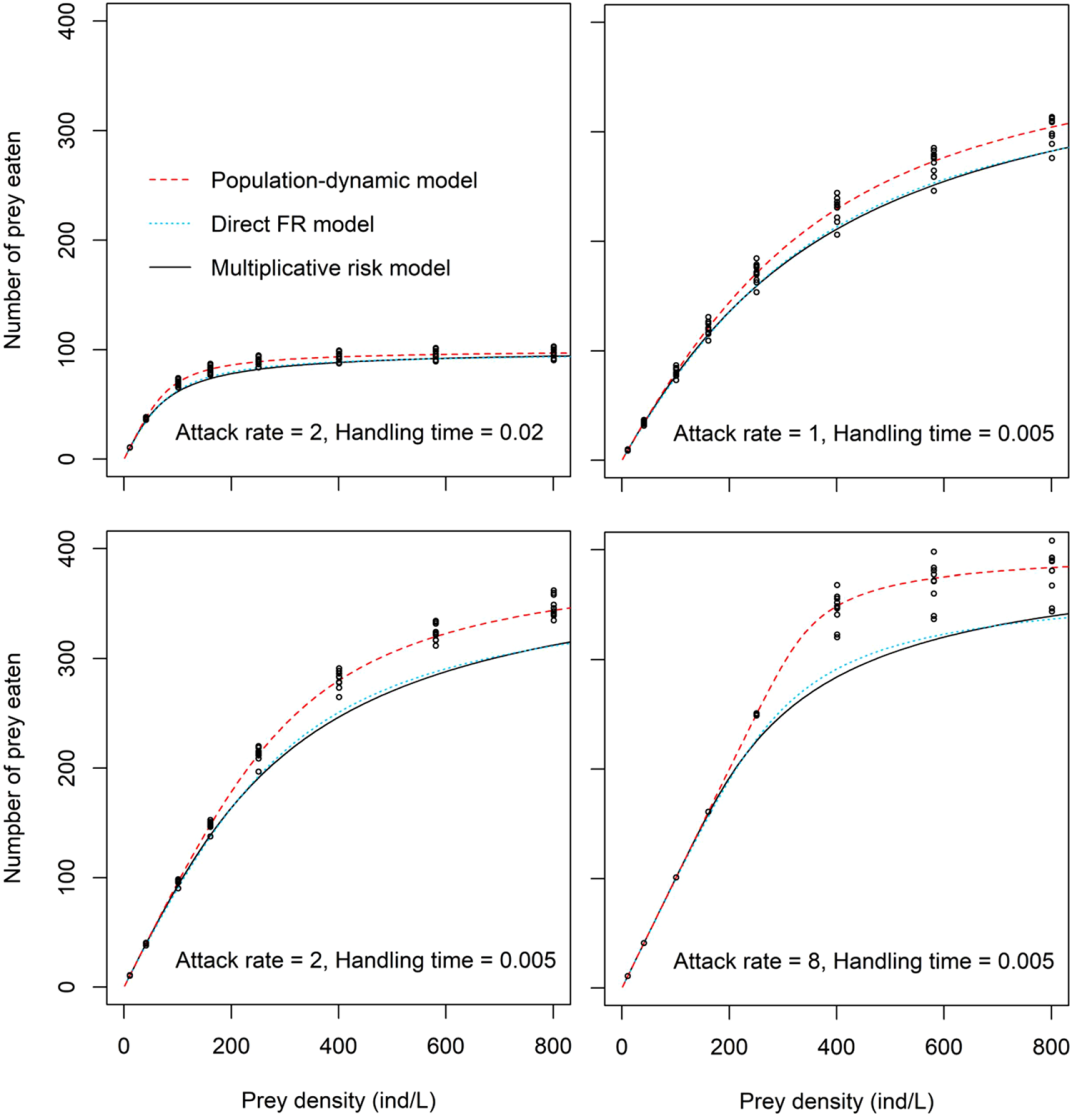
Simulated number of prey eaten over 24 hours by two conspecific predators under the assumption of independent predator effects on prey survival (black dots) for different values of attack rate and handling time. Lines represent predictions from the multiplicative risk model (solid black line), the direct FR model (dotted blue line) and the population-dynamic model (dashed red line). Range of values used for attack rate and handling time were chosen to reflect values found in previous studies conducting both a functional response experiment and multiple predator trials^10,20^.

When we compared the data from the simulated trials including two independent predators and the model predictions of each modelling approach for selected parameter values (Fig. 1), we found that the three modelling approaches captured well the non-linear relationship between prey density and the number of prey eaten by two predators. The population-dynamic model predictions also fitted well the simulated data, but the two other approaches (multiplicative risk model and direct FR model) tended to underestimate predation rates, especially for high attack rate and low handling time values. Moreover, predictions of the direct FR approach and the multiplicative risk model were nearly identical across the explored parameter space (Fig. 1).

**Figure 2:**
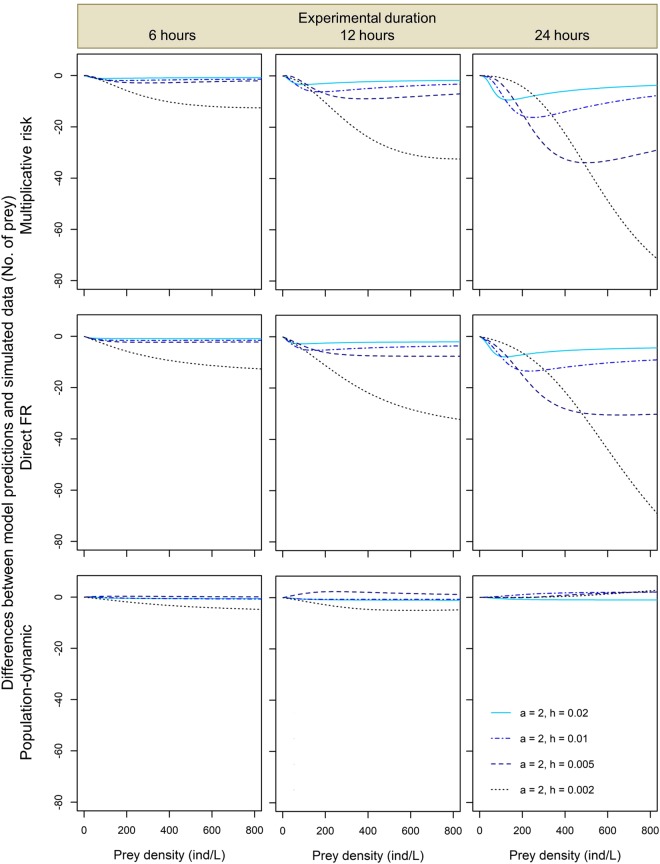
Pairwise differences expressed as differences in the number of eaten prey between model predictions and simulated data for two conspecific predators for the multiplicative risk model, the direct FR model and the population-dynamic model (top, middle and bottom row, respectively) in experiments lasting 6, 12 and 24 hours (left, middle and right column, respectively) for different values of handling time *h*. Attack rate *a* = 2 in all simulations.

**Figure 3:**
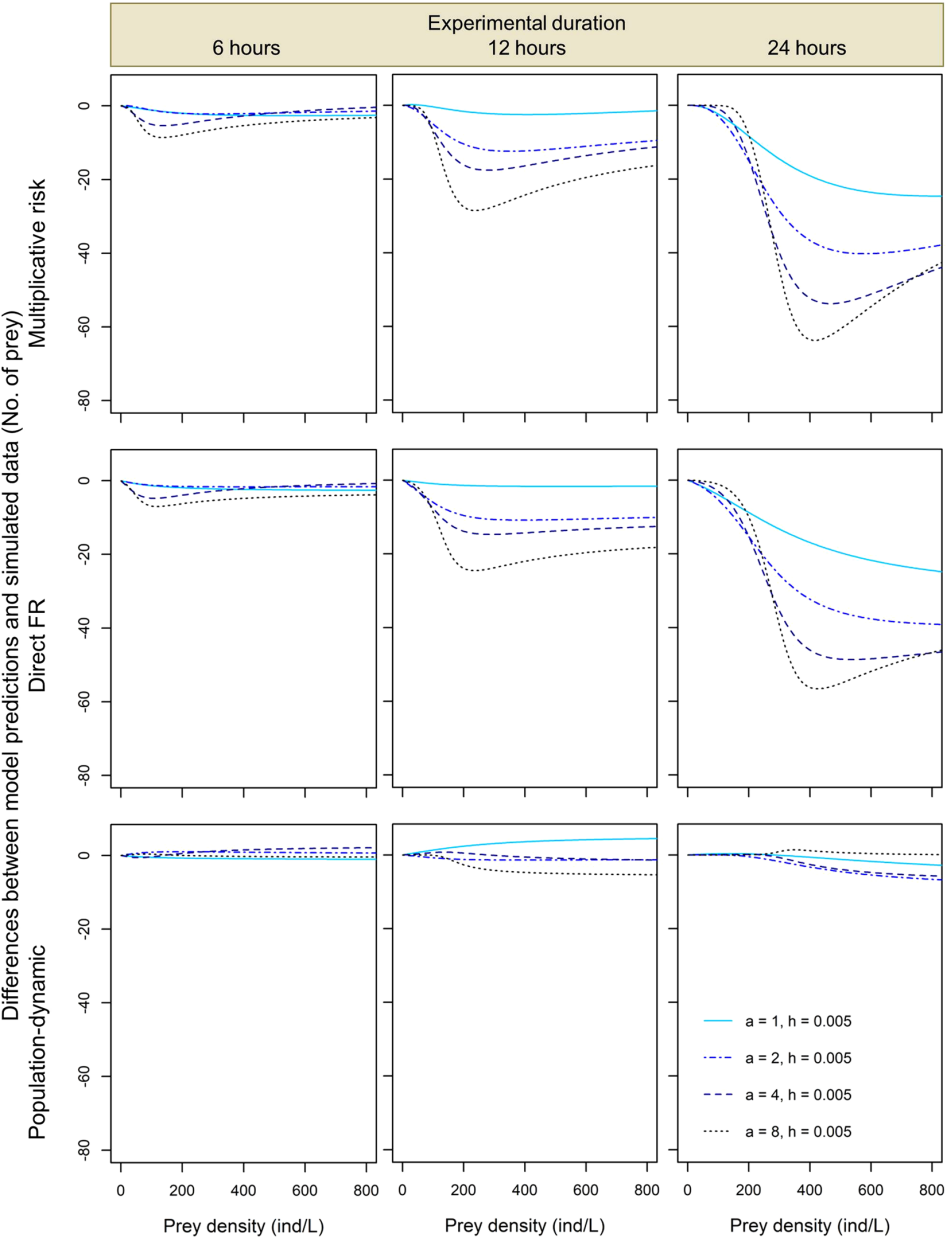
Pairwise differences expressed as differences in the number of eaten prey between model predictions and simulated data for two conspecific predators for the multiplicative risk model, the direct FR model and the population-dynamic model (top, middle and bottom row, respectively) in experiments lasting 6, 12 and 24 hours (left, middle and right column, respectively) for different values of attack rate *a*. Handling time *h* = 0.005 in all simulations.

**Figure 4:**
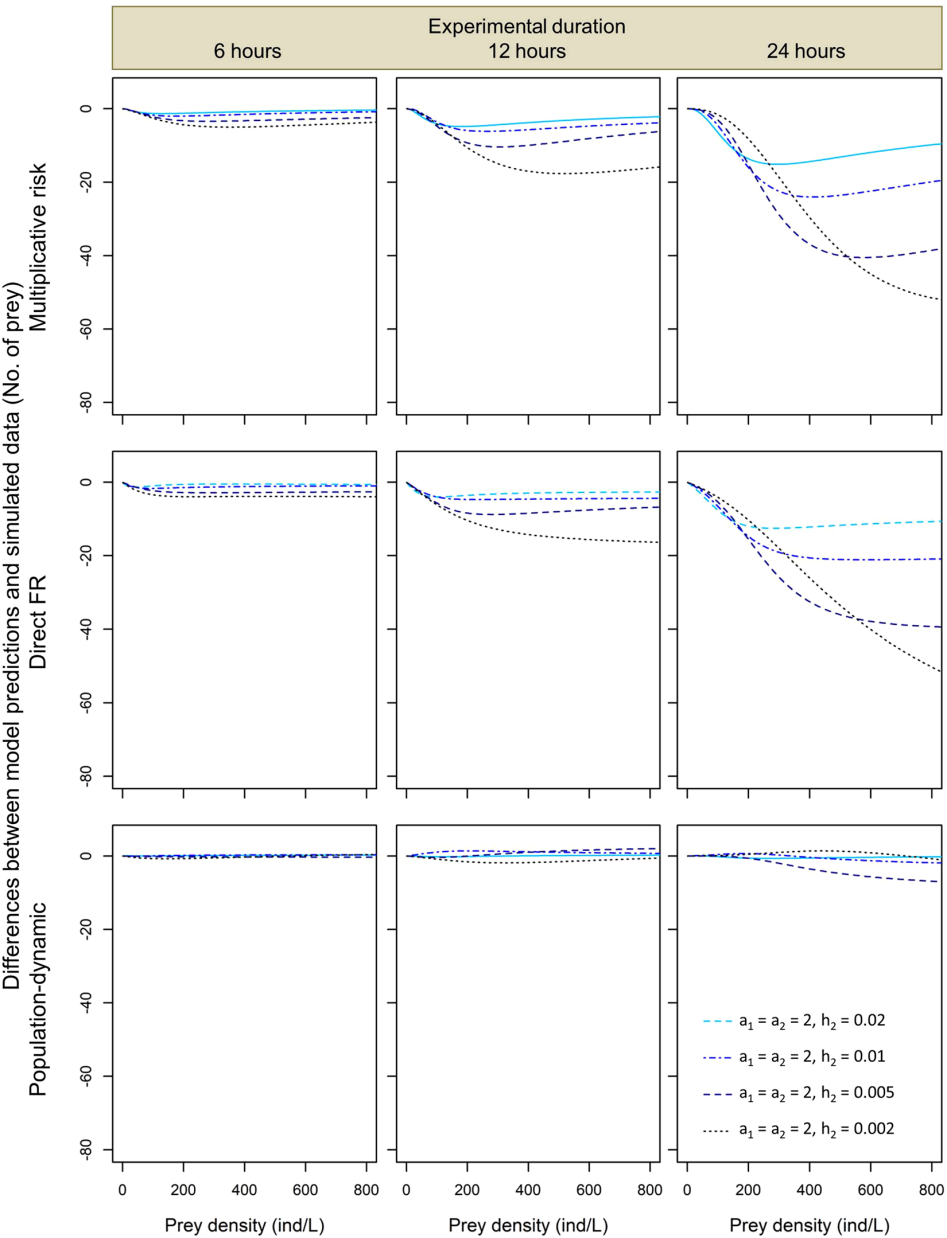
Pairwise differences expressed as differences in the number of eaten prey between model predictions and simulated data for two heterospecific predators for the multiplicative risk model, the direct FR model and the population-dynamic model (top, middle and bottom row, respectively), experimental time of 6, 12, or 24 hours (left, middle and right column, respectively), and different values of handling time *h*_2_ (see inside figure). Other functional response parameters (handling time *h* and attack rate *a*) fixed at *a*_1_ = *a*_2_ = 2 and *h*_1_ = 0.005.

**Figure 5:**
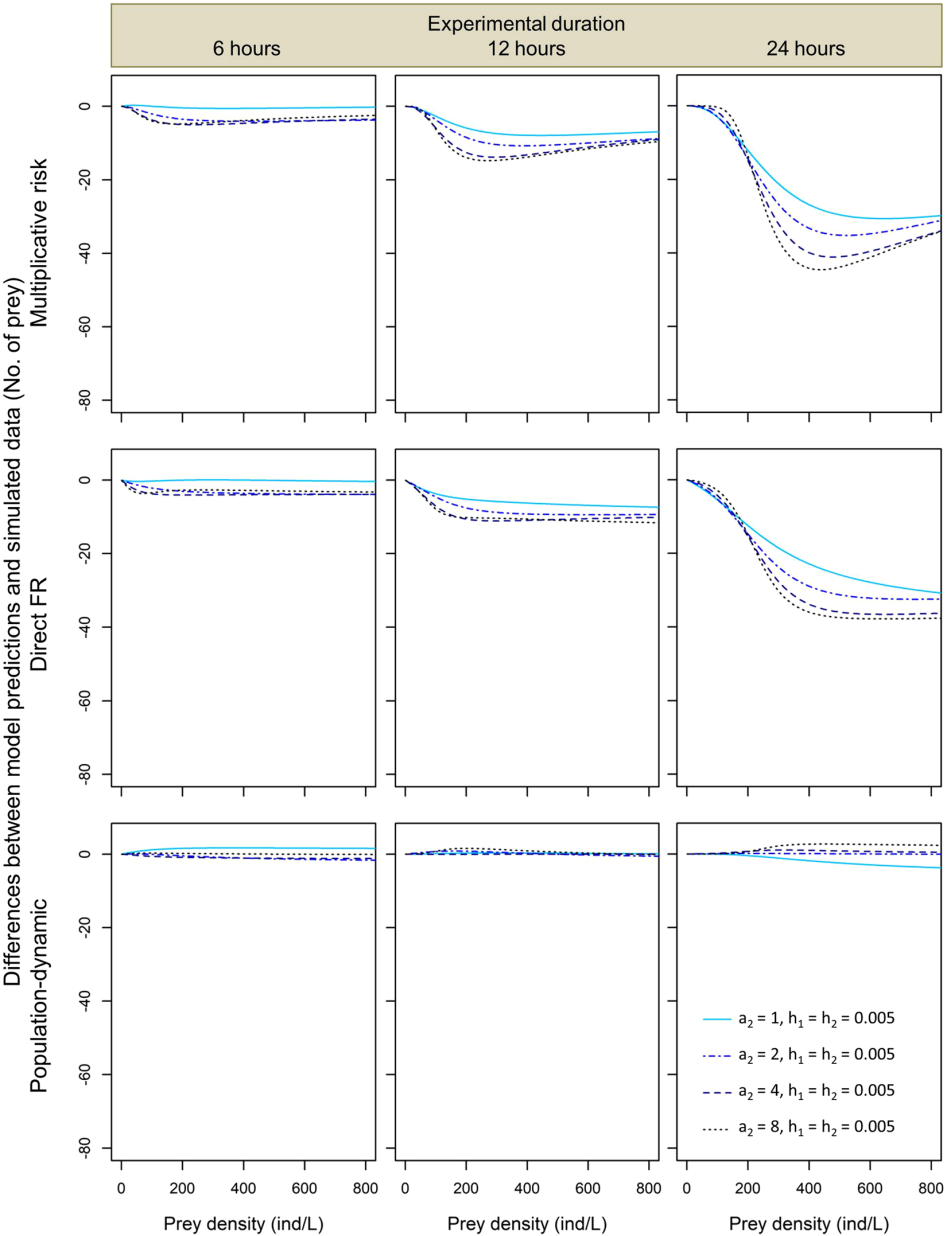
Pairwise differences expressed as differences in the number of eaten prey between model predictions and simulated data for two heterospecific predators for the multiplicative risk model, the direct FR model and the population-dynamic model (top, middle and bottom row, respectively), experimental time of 6, 12, or 24 hours (left, middle and right column, respectively) and different values of attack rate *a*_2_ (see inside figure). Other functional response parameters (handling time *h* and attack rate *a*) fixed at *a*_1_ = 2 and *h*_1_ = *h*_2_ = 0.005.

Further comparisons of model predictions to the simulated numbers of prey eaten across different values of the functional response parameters and experimental durations confirmed that the predictions of the direct FR approach and the multiplicative risk model tended to systematically underestimate predation rates by both conspecific (Figs 2 and 3) and heterospecific predators (Figs 4 and 5). Magnitude of this underestimation increased with experimental duration and predator attack rate *a*, decreased with handling time *h*, and either increased with or had a unimodal dependence on prey density in the studied range (Figs 2 and 3). As a result, the largest underestimations of predation rate were observed for 24-hour predation trials involving predators with the lowest values of handling time and high prey densities (Fig. 2). The lowest underestimations of predation rate were observed for 1-hour predation trials in which differences between model predictions and simulated data were negligible irrespective of the functional response parameter values and prey density (Fig. S1). Interestingly, underestimations tended to be less variable and less extreme for heterospecific than for conspecific predator assemblages. For instance, when handling time varied and attack rate remained constant we found that, at the highest prey density and 24-hour experimental duration, predation rate underestimations by the multiplicative risk model ranged from −3 to −71 for conspecific assemblages (Fig. 2) whereas they ranged from −10 to −51 prey for heterospecific assemblages (Fig. 4).

Unlike the multiplicative risk model and the direct FR model, the population-dynamic model predicted well the simulated numbers of prey eaten by two conspecific or heterospecific predators (Figs 2–5). Moreover, we found no systematic directional bias (over- or underestimation) for the predictions of the population-dynamic model across prey densities, functional response parameter values, experimental duration and predator assemblage type (conspecific or heterospecific). The population-dynamic model predictions sometimes diverged from the simulated data at high prey densities, but the difference was usually minor and much smaller than in the two other modelling approaches (Figs 2–5).

## Discussion

Non-independent interactions among predators can have important consequences for the structure and dynamics of ecological communities^10,13,27^. For instance, interference among predators decreases *per capita* trophic interaction strengths which, in turn, can increase community stability and dampen the destabilizing effect of resource enrichment^28^. Quantifying emergent MPEs and determining when and how they change along ecological gradients (e.g., resource density) is thus central for a full understanding of the effects of natural and anthropogenic pressures on food web structure and dynamics^6,9–11,13,17^. As the quantification of MPEs relies on the comparison of empirical observations (prey survival under predation by multiple predators) with predictions from a model assuming independent predator individual effects, the accuracy and robustness of these predictions are crucial to assess the sign and magnitude of MPEs. In this study, we investigated the strengths and limitations of three modelling approaches (the popular multiplicative risk model, the direct FR model, and the population-dynamic model) and compared their predictions using simulated data sets. Our findings emphasize the importance of accounting for the non-linearity and time-dependence of species interactions to improve the detection and quantification of emergent multiple predator effects.

### Model comparisons based on simulated data

The multiplicative risk model proposed by Soluk^18^ has been used extensively in the ecological literature to quantify MPEs. However, McCoy *et al*.^13^ showed that this model can lead to false positives and detect MPEs even when predators affect prey independently. Our results based on simulated data corroborate those of McCoy *et al*.^13^ by showing that the multiplicative risk model tends to underestimate predation rates, especially when experimental duration is long, prey density is high and when the predators have short handling times and high attack rates (top rows in Figs 2–5). We also show that these qualitative conclusions apply to both conspecific and heterospecific predators (Figs 2–5).

Surprisingly, we have found that the predictions of the direct FR model and the multiplicative risk model are very similar for most of the explored parameter space. As a result, the systematic biases of the multiplicative risk model described above apply to the direct FR model as well: we found that this model tends to underestimate simulated predation rates by two conspecific or heterospecific predators (middle rows in Figs 2–5) except for very short experiments (Fig. S1). In the direct FR approach, a functional response model is fitted to the expected numbers of prey eaten by multiple predators. These expected numbers are generated using the multiplicative risk model, which explains the closely matching predictions of the two approaches. Based on our simulated data, we thus conclude that the direct FR approach suffers from similar biases as the multiplicative risk model and can only be used when prey depletion is negligible and predators handle and digest prey rapidly (i.e., handling time is negligible). These restrictive conditions are likely to be violated in most experimental and natural systems as prey depletion is often substantial^11,21^ and most predators have a nonlinear type II functional response (i.e., non-negligible handling times)^26^.

Moreover, the magnitude of the underestimation of the number of prey eaten in heterospecific predator assemblages produced by the multiplicative risk model and the direct FR model generally lie between the magnitudes observed in the two conspecific assemblages of these predators (Figs 2 and 3 vs Figs 4 and 5). This difference is linked to the fact that one of the two heterospecific predators has fixed handling time and attack rate values that are not at the limit of the range used in our simulations. In other words, the likelihood of having two predators with extreme values of handling time or attack rate is lower for heterospecific than for conspecific predator assemblages, which explains why model biases are less extreme in the former assemblage type.

To overcome the biases of the multiplicative risk model, McCoy *et al*.^13^ proposed the population-dynamic model as an alternative approach based on functional response modeling that accounts for prey depletion and non-linear feeding rates. In a simulation study, they showed that the population-dynamic model produces unbiased predictions of the independent effect of predators on prey survival. In line with the results of McCoy *et al*.^13^, we found that the population-dynamic model predicts well the number of prey eaten by two conspecific or heterospecific predators (bottom rows in Figs 2–5). Although predictions of this model were less accurate at high prey densities, we found no systematic and substantial bias in the predictions of the population-dynamic model. This indicates that the predictions are robust against variations in functional response parameter values, experimental duration and composition of predator assemblages. Overall, we found that the population-dynamic model is more accurate and less biased than the multiplicative risk model and the direct FR model. We thus recommend using the former to predict the expected number of prey eaten by multiple predators under the assumption of predator independent effects.

### Implication for the detection and quantification of MPEs

As mentioned in the Introduction, MPEs occur when the predictions based on single-predator feeding trials do not match observations from feeding trials involving multiple predators. The latter combine antagonistically (e.g., through predator interference) and synergistically (e.g., through predator facilitation) when the predictions overestimate and underestimate the observations, respectively. Our results indicate that the application of the multiplicative risk model and the direct FR model will likely lead to false positives (i.e., identify predators as synergistic) in the absence of MPEs because they systematically underestimate predation rates by multiple predators. This further implies that these two modelling approaches are less likely to detect antagonistic predator effects.

Even when the multiplicative risk model and the direct FR model get the MPEs qualitatively right, the two approaches will systematically overestimate synergistic predator effects and underestimate antagonist predator effects, especially at high prey densities or under more extensive prey depletion. Extensive prey depletion arises when predators have short handling time, high attack rates, or in longer experiments. Prey depletion is non-negligible in most predation studies^21^ that last sufficiently long and do not replace eaten prey. Since 67% of the functional response experiments published so far were conducted over a 24-hour period and usually did not replenish eaten prey^29^, we conclude that the predictions of the direct FR model and multiplicative risk model will be unreliable in ‘typical’ MPE studies. In contrast, the population-dynamic model is more accurate and does not suffer from systematic biases. As a result, this model is much less likely to detect false positives (i.e., presence of MPEs) and the latter will not suffer from systematic biases towards synergetic or antagonistic effects.

Altogether, our results show that the detection and quantification of MPEs depends on the modelling approach used to predict the expected number of prey eaten by multiple predators. The choice of the modelling approach to detect MPEs can thus have serious consequences for our understanding of species interactions in complex food webs involving multiple predators.

### Model assumptions, biological interpretation of parameters, and possible limitations and extensions

Functional response is a widely used mechanistic approach to model feeding rates, but its application beyond single consumers is not without problems and the need for careful and critical application has been emphasized repeatedly^25,30,31^. Functional response is a *per capita* rate, or in a more general sense, consumption rate per unit of consumer^30^. In other words, the functional response parameters (attack rate and handling time) are defined per individual predators. Their estimates from a single-predator functional response model fitted to multi-predator assemblages, as in the direct FR approach, lack mechanistic meaning and cannot be interpreted beyond providing a statistical fit to the data. In other words, these parameter estimates are aggregated attack rate and handling time values of a single predator, multiple conspecific predators or multiple heterospecific predators. They do not directly account for predator density or diversity and are thus incomparable across treatments. This prevents a meaningful interpretation of the functional response parameters and hampers the spatial or temporal extrapolation of the results, including the development of population-dynamic and food web models based on estimated functional response parameters.

To circumvent the limitations described above, the population-dynamic approach uses estimates of functional response parameters from single-predator feeding experiments to parameterise a simple population-dynamic model. These estimates are defined per predator, have a meaningful and mechanistic interpretation, and one can collect other functional parameter estimates from the literature (or from another experiment) to predict feeding rate by multiple predators using the population-dynamic approach, which is not possible with the direct FR approach or the multiplicative risk model. Moreover, the population-dynamic approach takes into account the non-linearity of feeding rates and prey depletion unlike the multiplicative risk model and predictions derived from it^10,13^. The population-dynamic approach can also cover situations in which the functional response shapes differ among predators (e.g., when one has a type II and another type III response), which goes beyond the direct FR approach. For these reasons, the population-dynamic approach can be applied to more complex food-web models and longer timescales by, for instance, including prey and predator reproduction and tracking of population cycles.

The main limitation of the population-dynamic model compared to the multiplicative risk model is that the former requires estimating the functional response parameters of each predator species. This implies conducting single-predator feeding trials along a gradient of prey density, which can be time consuming and labour intensive. Using the multiplicative risk model requires single-predator feeding trials only for the experimental prey densities at which multiple-predator trials are conducted, which can make it much less labour intensive when the multiple-predator trials are conducted over only one or a few prey densities. The direct FR approach combines the limitations of both former approaches: it requires the same amount of data as the population-dynamic model but suffers from similar biases as the multiplicative risk model. We conclude that the most accurate approach is the population-dynamic model but if experimental workload is severely limiting, one may consider trading the workload against accuracy by using the multiplicative risk approach.

## Conclusions

Developing a robust and accurate modeling approach to quantify MPEs is crucial for advancing the study of MPEs and improving the realism of predator-prey and food-web models. In this study, we found that the predictions of the direct FR model and the multiplicative risk model are very similar and systematically underestimate predation rates by multiple predators at higher prey densities or when prey depletion is substantial, especially when experimental trials last longer than a few hours. Moreover, the functional response parameters estimated with the direct FR approach lack mechanistic interpretation, which limits the understanding of the mechanisms driving multiple predator interactions and prevents extensions of the direct FR approach to more complex food web structure or dynamics. Although it requires estimating the functional response of each predator species, our study indicates that the population-dynamic approach is more robust, precise, and informative than the multiplicative risk model and the direct FR approach. We thus recommend using the population-dynamic approach in future MPEs studies. In particular, studies using predators with different functional response shapes as well as experiments with substantial prey depletion should provide a critical test to the wider applicability of the population-dynamic approach to MPE studies.

## Material and Methods

### The direct FR approach

This approach proposed by Wasserman *et al*.^20^ consists of three main steps: (1) application of the multiplicative risk model (eqn 1) to data from single-predator trials to generate expected numbers of prey eaten by multiple predator, (2) fits of the expected data generated in the previous step and of data observed in multiple-predator trials with a functional response model, and (3) comparison of the fits based on the overlap of the confidence intervals (CIs) of both functional responses.

In the first step, their approach is to randomly assign a replicate number to each trial of the single-predator experiment and use these replicate numbers to generate predictions for multiple-predator treatments with the multiplicative risk model (eqn 1). For instance, replicate number 1 of a predator A feeding on a given prey density is associated with replicate number 1 of a predator B feeding on the same prey density to predict (using eqn 1) the combined predator A-predator B predation rate at the given prey density. For pairs of conspecific predators, their approach is to use the same replicate twice (note that this leads to pseudoreplication issues).

In the second step, after determining the functional response type (II or III) using standard methods^25^, the data are fitted with the Rogers’ random predator equation^32^ that accounts for prey depletion during the time course of the experiment:

2 $${N}_{e}={N}_{0}(1-\exp (-a(t-h{N}_{e})))$$

where *N*_e_ is the number of prey eaten, *N*_0_ is the initial prey density (prey.arena^−1^), *t* is the duration of the experiment (day), *h* is the prey handling time (day.prey^−1^), and *a* is the predator attack rate (arena.day^−1^), which is constant for type II and a function of *N*_0_ for a type III functional response. Two separate instances of eqn 2 are fitted using a maximum-likelihood method; one to the predicted values for multiple-predator treatments generated at step 1 and another to the observed data from the multiple-predator trials. Non-integer predictions that often arise from the multiplicative risk model (eqn 1) need to be rounded to the nearest integer, because fitting eqn 2 using a maximum-likelihood method with a binomial distribution requires integer data^31^.

In the third step, datasets are non-parametrically bootstrapped (n = 2000 in the study by Wasserman *et al*.^20^) and eqn 2 is fitted to each bootstrapped dataset using starting values of the functional response parameters (*a* and *h*) that were obtained from the original maximum likelihood estimates (step 2). The bootstrapped fits are then used to construct 95% CIs around functional response curves using the “frair” package^33^. Finally, functional response curves are visually compared based on the overlap of their 95% CIs; non-overlapping CIs are classified as significant differences between the functional response curves^33^.

### The population-dynamic approach

The approach proposed by McCoy *et al*.^13^ also consists of three main steps: (1) estimation of functional response parameters from single-predator trials, (2) use of these parameter estimates to predict the expected predation rate by multiple predators using a population dynamic model and (3) comparison of these predictions to the observed empirical values.

In the first step, after determining the functional response type (II or III) using standard methods^25^, functional response parameters of each predator species are estimated by fitting eqn 2 to the single-predator experimental data using a maximum-likelihood method with a binomial distribution^31^.

In the second step, McCoy *et al*.^13^ proposed to use functional response parameter estimates from single-predator experiments (obtained in the first step) in a population-dynamic model of prey depletion by multiple predators in time:

3 $$\frac{dN}{dt}=-{{\sum }_{i=1}^{n}{f}_{i}(N)P}_{i}$$

where *N* is the prey population density, *P*_*i*_ (*i* = 1, 2, …, *n*) are the population densities of predators *i* and *f*_*i*_(*N*) is the functional response of predator *i*. Equation 3 can accommodate any functional response shapes as, for instance, the Holling type II functional response, *f*_*i*_(*N*) = *a*_*i*_ *N*/(1 + *a*_*i*_ *h*_*i*_ *N*), which includes the estimates of prey handling times *h*_*i*_ (day.prey^−1^) and attack rates *a*_*i*_ (arena.day^−1^) of predators *i* obtained in the first step. This “null” model assumes no emergent MPEs and its predictions can be compared to multiple-predator feeding experiments to assess the sign and strength of MPEs. To generate predictions of expected prey survival in the multi-predator experiments, initial values of *N*_0_ and *P*_*i*_ (*i* = 1, 2, …, *n*) are set at the experimental initial prey and predator densities corresponding to the experimental treatment and the population dynamic model given by eqn 3 is integrated over the time interval of the experiment. The lower and upper 95% CIs around the predictions are estimated with a global sensitivity analysis that uses the 95% CIs of each functional response parameter estimate and their variance-covariance matrix (covariance is assumed to be zero when unknown) to generate a number of random parameter sets using a Latin hypercube sampling algorithm^34^. For each parameter set, eqn 3 is then integrated over time and expected prey survival calculated using, e.g., the’sensRange’ function in the R package’FME’^34^. The 2.5% and the 97.5% quantiles of the survival values obtained from these simulations are used as the lower and upper 95% CIs around the prediction.

For the third step, predictions of the population dynamic model are compared to the observed values based on the overlap of their 95% CIs for each predator treatments.

### Simulated data

We simulated predation trials by numerically solving the population dynamic model (eqn 3) with a nonlinear type II functional response, which is the most common type of predator functional response^26^. We used two types of simulations: deterministic to generate predictions of the three models based on single-predator trials, and stochastic to simulate ‘realistic’ trials including two predators to which we compared the model predictions.

In the deterministic simulations, we first generated data for single-predator trials (characterized by their species-specific handling time and attack rate values) along a gradient of prey density ranging from 1 to 800 prey.L^−1^. For the functional response parameters used in the simulations, this prey range covers both the increasing and the asymptotic part of the functional response and covers a realistic range of prey densities such as the densities of *Daphnia* in fishless ponds in Central Europe (Sentis *et al*. unpublished data). We then (1) used these simulated data to feed the multiplicative risk model and predict cases with two predators under the assumption of pairwise independent effects, (2) fitted the functional response model to the predictions from the multiplicative risk model (i.e., the direct FR approach) and (3) used the population-dynamic model to generate predictions with two predators using the same set of parameters as the one used to generate data for the predator monoculture. Altogether, these different steps resulted in generating three predictions (one for each modelling approach) for each prey density about the expected multiple predator effects on prey survival under the assumption of predator independence, i.e., no emergent effects beyond prey depletion.

To estimate how well the different models can predict consumption rate by multiple predators under the assumption of predator independence, we used a stochastic approach to simulate predation trials for two conspecific or heterospecific predators by numerically solving eqn 3 using the same set of parameters as the one used to generate data for the predator monoculture. To mimic realistic experimental data and account for uncertainty in functional parameter estimates, we assumed that the parameters of the functional response, the handling time *h* and the attack rate *a*, are normally distributed with a standard deviation of respectively 5 and 10% of the mean parameter value reflecting typical parameter uncertainty values found in functional response experiments^10,35^. To achieve this, we allowed values of parameters *a* and *h* to vary (following the normal distribution specified above) at each time step of the numerical integration of eqn 3, which generated variation in the simulated data. Eqn 3 was integrated 10 times for each set of parameters values to mimic empirical functional response data sets that often include up to 10 replicates per prey density^10,20,35,36^. This yielded 10 replicates of simulated data (using 5 or 15 replicates did not substantially alter our conclusions, see Figs S2, S3).

We first illustrated the data for simulated trials including two predators and the predictions of the different modeling approaches for a selection of parameter values (see Fig. 1). We then systematically quantified pairwise differences between model predictions and the simulated data for two predators as the expected number of prey eaten (model prediction) minus the simulated number of prey eaten (mean of the 10 replicates). This yielded three pairwise differences, one between the multiplicative risk model and the simulated data, one between the direct FR approach and the simulated data, and one between the population-dynamic model and the simulated data. We used these differences as a measure of bias in the three modelling approaches; note that negative differences correspond to a situation in which the model predicts lower prey consumption by two predators than observed in the simulated trials.

We then determined how these differences changed with study duration *T*, handling time *h*, attack rate *a* and prey density *N*, all of which influence depletion and/or the nonlinearity of the functional response^13,24,25,32^. We used two predators (*P*_1_ = *P*_2_ = 1) and varied *T* from 1 to 24 hours in all simulations, which covers most of the range of experimental durations over which functional response experiment are conducted^29^. We first assumed two conspecific predators (i.e., *a*_1_ = *a*_2_ = *a* and *h*_1_ = *h*_2_ = *h*) and varied *h* from 0.002 to 0.02 while keeping *a* = 2. We next varied *a* from 1 to 8 while keeping *h* = 0.005. We then explored scenarios with two heterospecific predators with a fixed handling time and attack rate values of the first predator (*a*_1_ = 2 and *h*_1_ = 0.005). We first varied *h*_2_ from 0.002 to 0.02 while fixing the attack rate of the second predator (*a*_2_ = 2) and then varied *a*_2_ from 1 to 8 while fixing *h*_2_ to 0.005. The range of values used for *a* and *h* were chosen to reflect values found in previous studies conducting both functional response experiment and multiple predator trials^10,20^.

Simulations were performed with R version 3.4.1^28^ using the package ‘bbmle’^31^ to fit Roger’s random predator equation and the packages ‘deSolve’^37^ and ‘FME’^34^ for the population dynamic model simulations.

### Data accessibility statement

We confirm that the R code supporting the results will be archived in an appropriate public repository such as Dryad or Figshare.

## Electronic supplementary material

Supplementary information
