# Supplementary material for: On the use of functional responses to quantify emergent multiple predator effects
Source: Sci Rep. 2018 Aug 6;8:11787. doi: 10.1038/s41598-018-30244-9 (PMC6079024; doi:10.1038/s41598-018-30244-9)
Supplement: Supplementary file 1 — Supplementary information [file 41598_2018_30244_MOESM1_ESM.docx]

**Supplementary information**

**On the use of functional responses to quantify emergent multiple predator effects**

Arnaud Sentis and David S. Boukal

**Figure S1.** Pairwise differences expressed as differences in the number of prey eaten between model predictions and simulated data for two conspecific predators for the multiplicative risk model, the direct FR model and the population-dynamic model (top, middle and bottom row, respectively) and different values of handling time *h* (left column) and attack rate *a* (right column) in experiments lasting 1 hour.


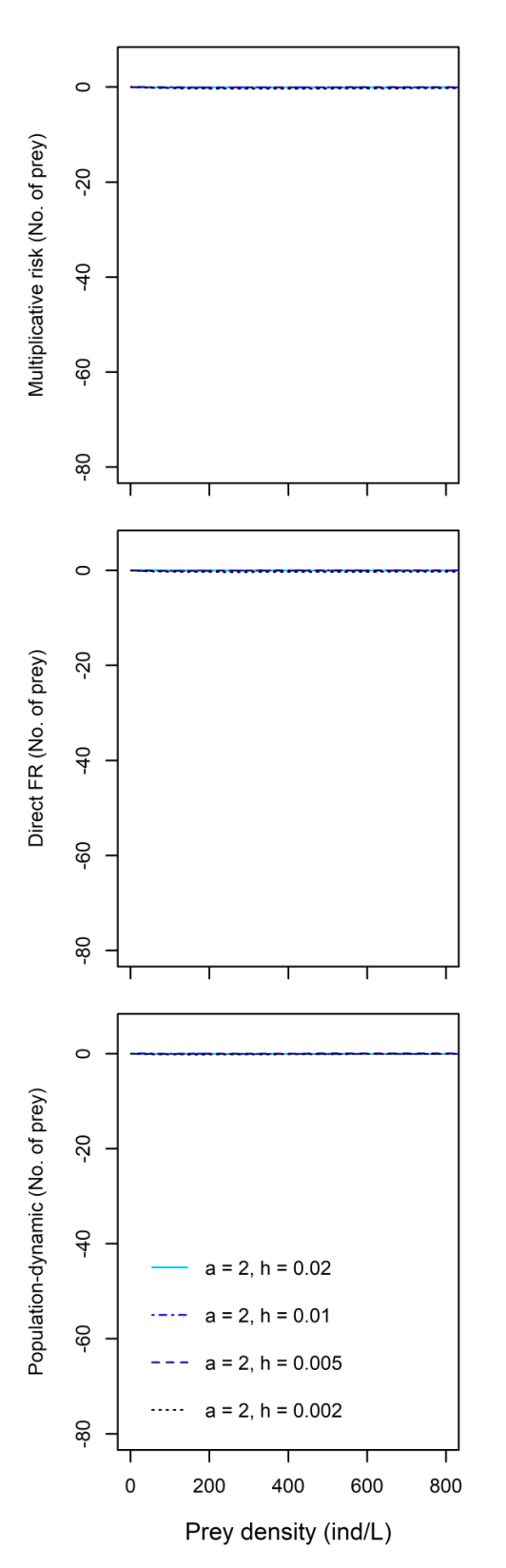

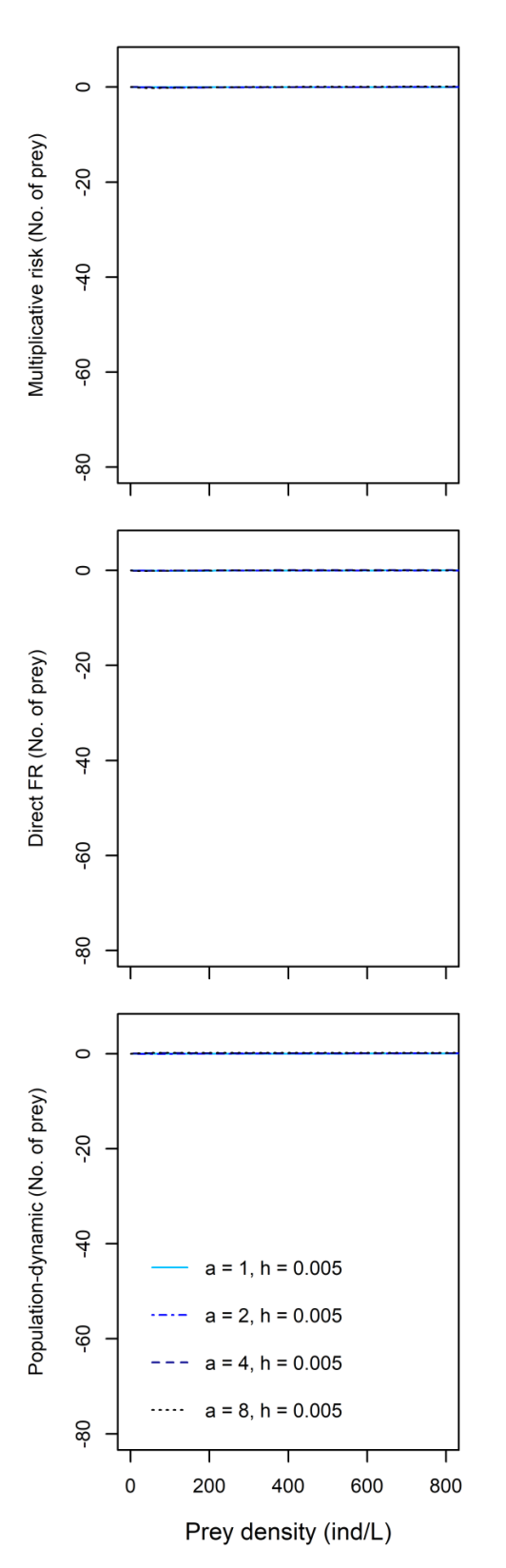


Differences between model predictions and simulated data (No. of prey)

Population-dynamic Direct FR Multiplicative risk

**Figure S2.** Pairwise differences expressed as differences in the number of prey eaten between model predictions and observations for two conspecific predators for the multiplicative risk model, the direct FR model and the population-dynamic model (top, middle and bottom row, respectively) for different values of handling time *h* and different number of replicates per prey density (left column: 5, middle column: 10 replicates, and right column: 15 replicates) in experiments lasting 24 hours.

Number of replicates in the simulated data

5 10 15


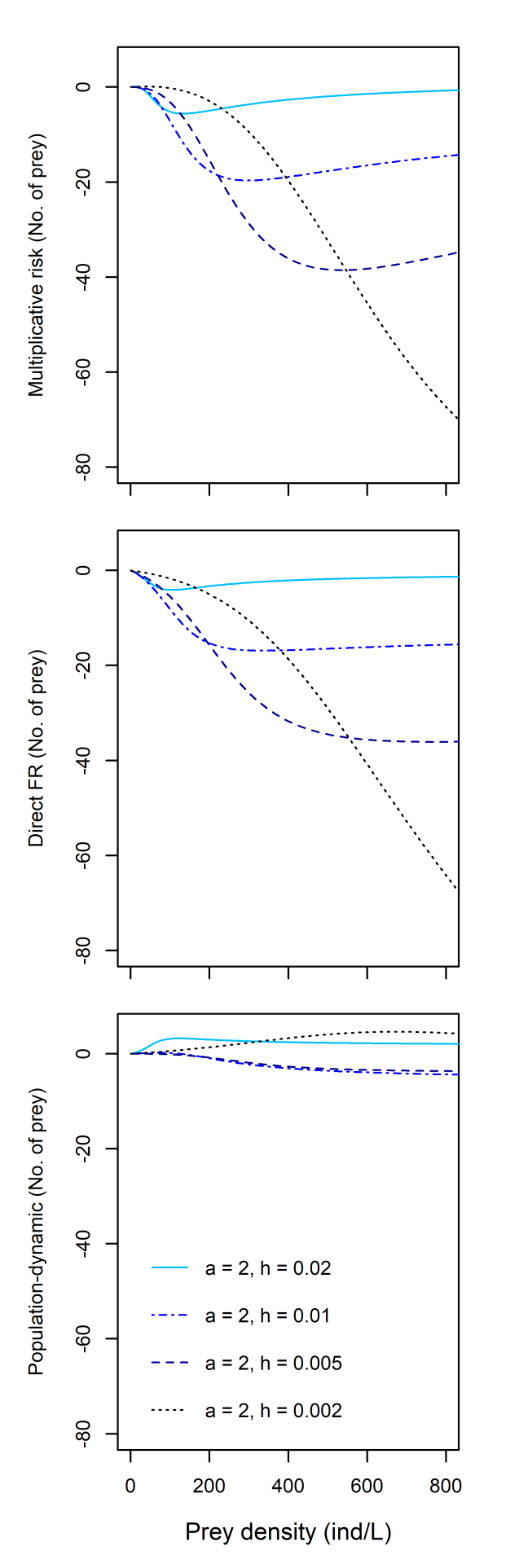

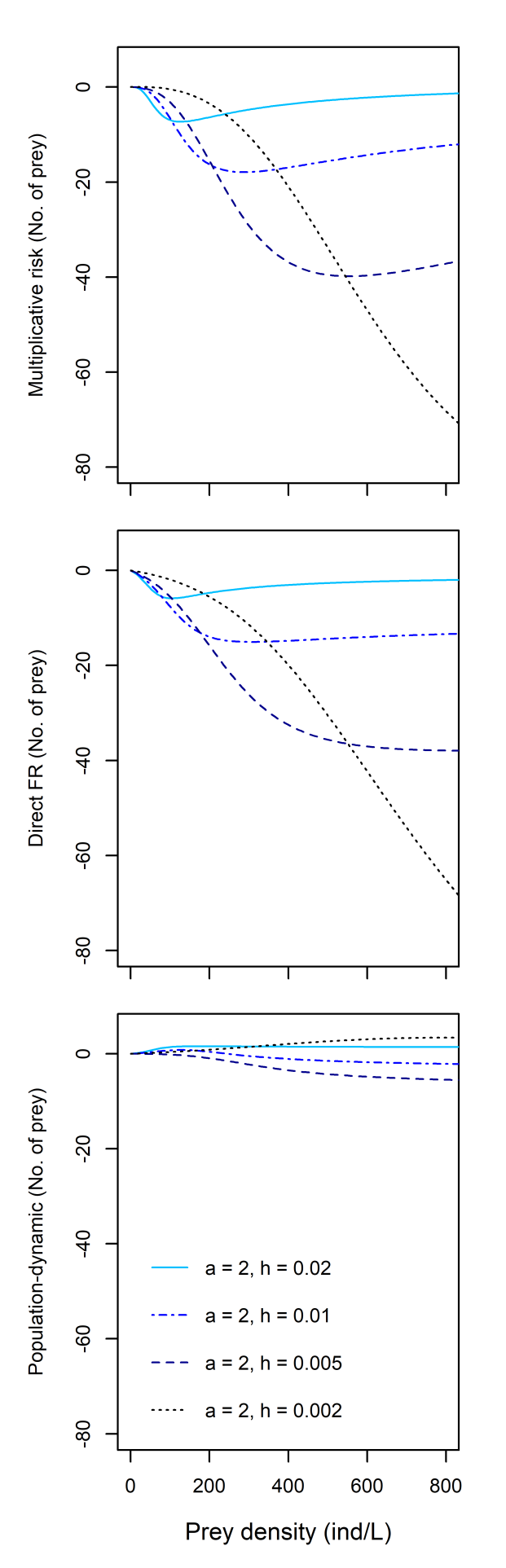

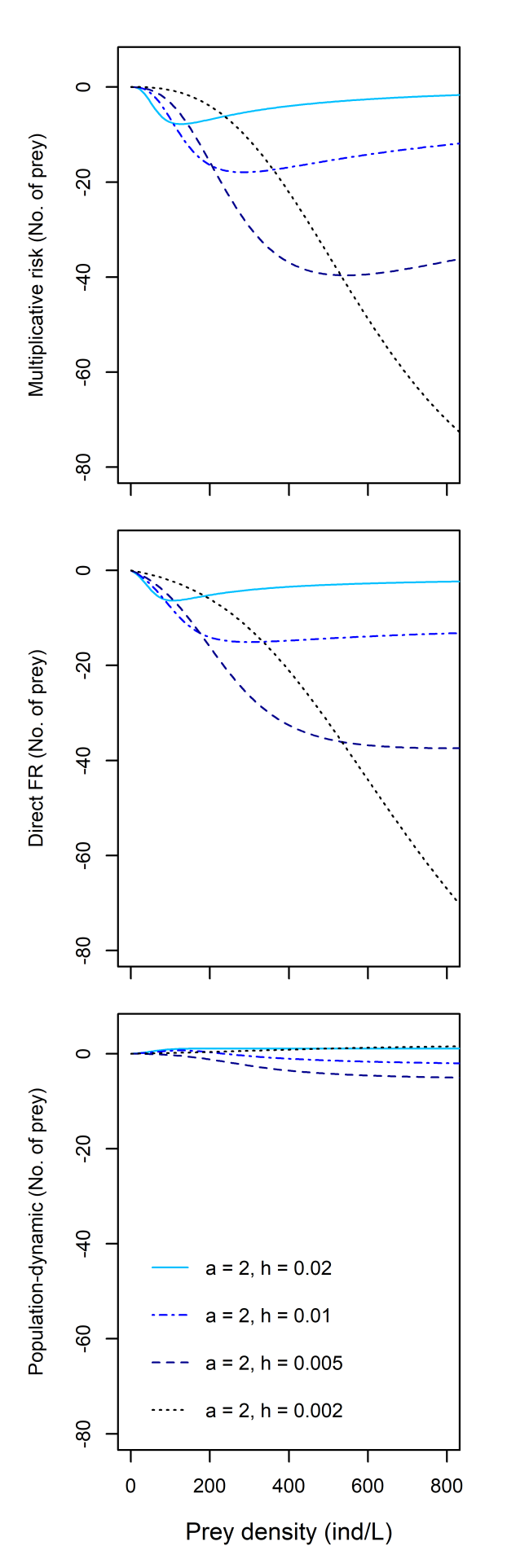


Differences between model predictions and simulated data (No. of prey)

Population-dynamic Direct FR Multiplicative risk

**Figure S3.** Pairwise differences expressed as differences in the number of prey eaten between model predictions and observations for two conspecific predators for the multiplicative risk model, the direct FR model and the population-dynamic model (top, middle and bottom row, respectively) for different values of attack rate *a* and different number of replicates per prey density (left column: 5 replicates, middle column: 10 replicates, right column: 15 replicates) in experiments lasting 24 hours.

Number of replicates in the simulated data

5 10 15


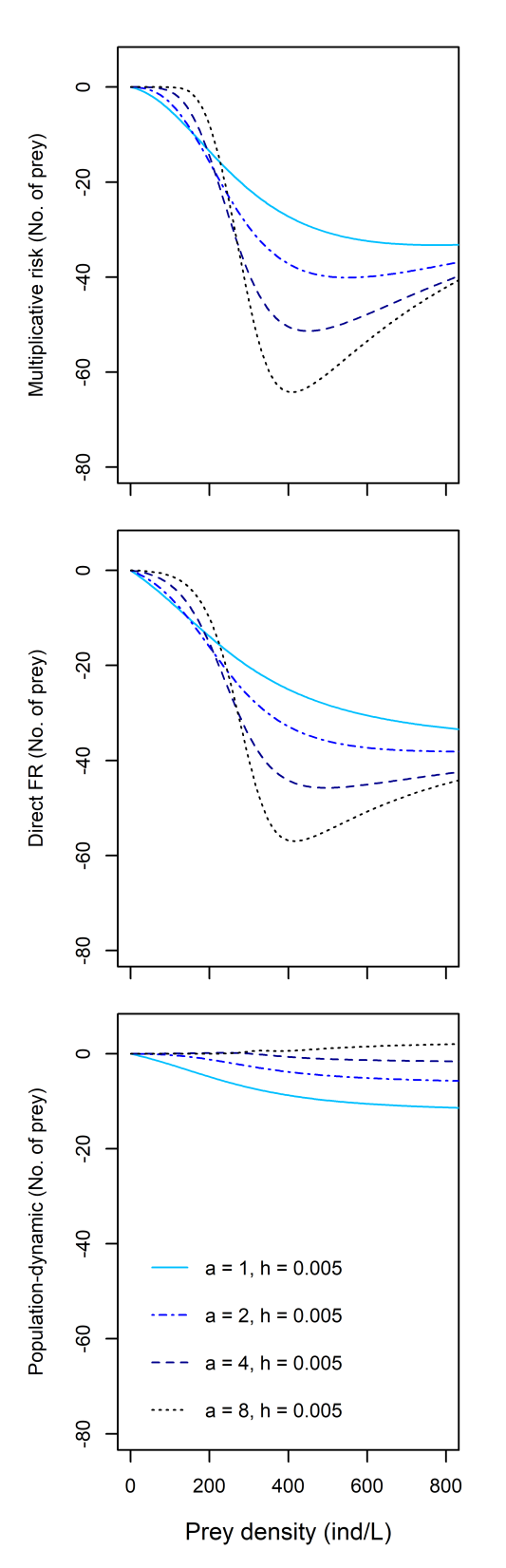

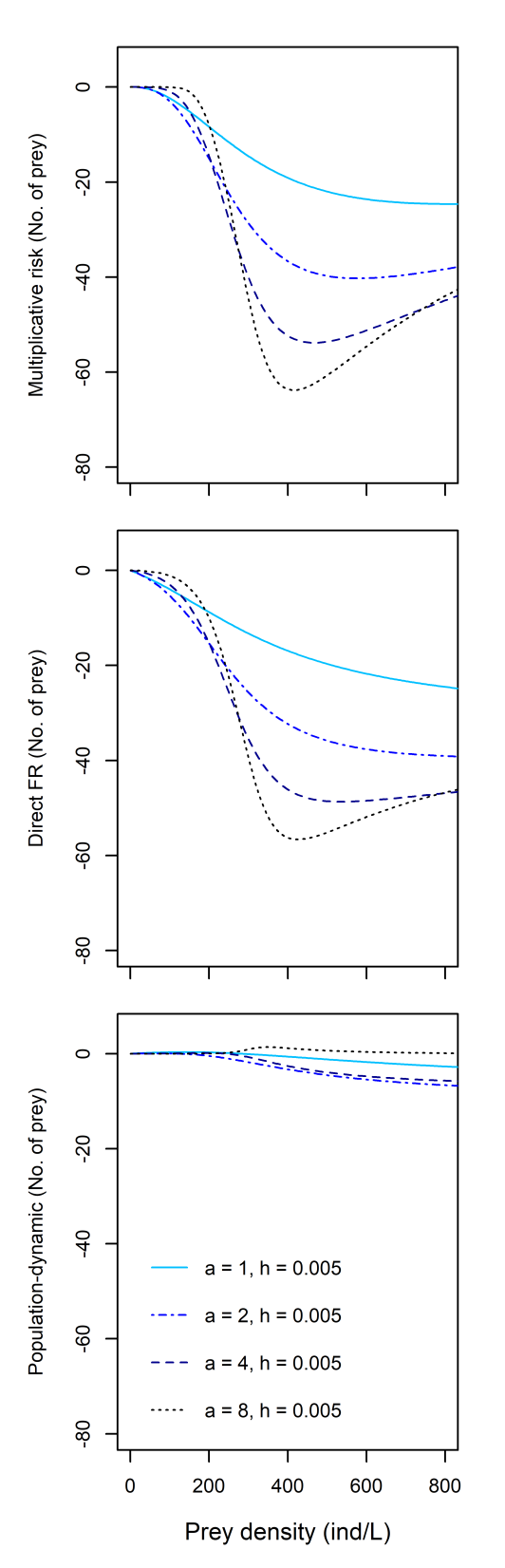

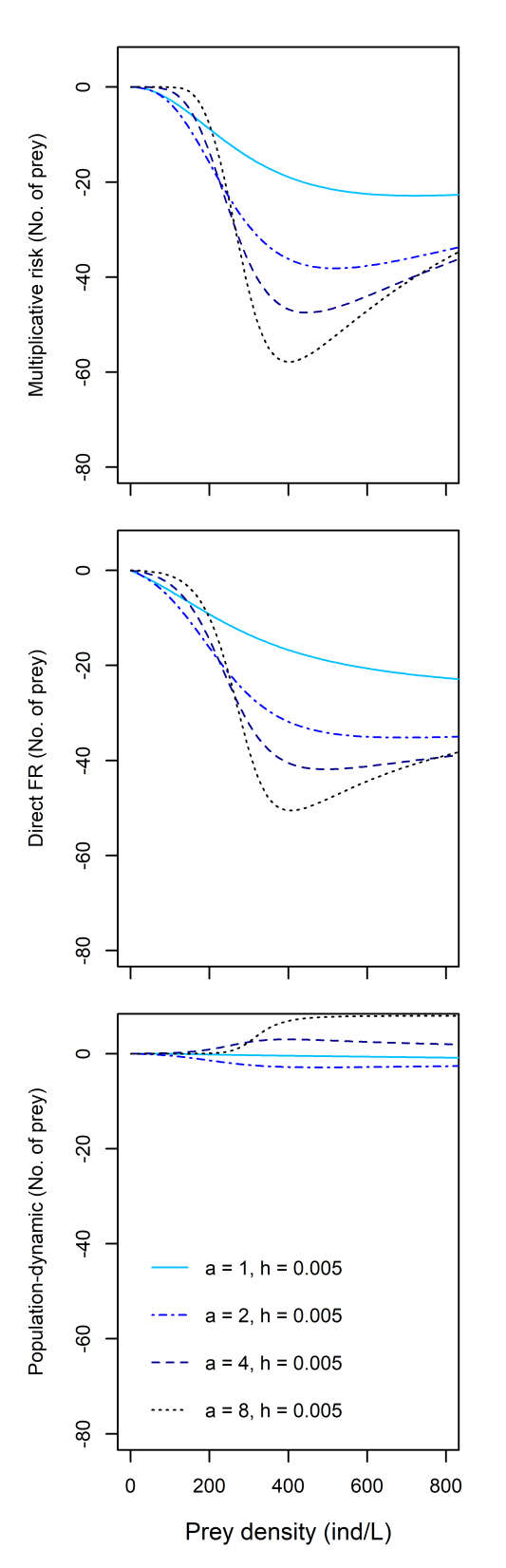


Differences between model predictions and simulated data (No. of prey)

Population-dynamic Direct FR Multiplicative risk
